# Just Closed

**Published:** 1882-03

**Authors:** 


### JUST CLOSED.

The sessions of the thirty-seventh annual meeting of the Mississippi Valley Dental Society have just closed. The occasion has been one of unusual interest. Excellent papers, and instructive discussions were the order of the occasion. An interesting feature was the large number of the younger members of the profession present, and the interest they took in the proceedings.

The papers, discussions, and proceedings will appear in the April and May numbers of the REGISTER, which the readers of the REGISTER may read with profit.

